# Glimpse Into the Hemodynamic Performance of Myval Series vs Sapien 3 Ultra Resilia

**DOI:** 10.1016/j.shj.2024.100394

**Published:** 2025-01-17

**Authors:** Akihiro Tobe, Yoshinobu Onuma, Osama Soliman, Andreas Baumbach, Patrick W. Serruys

**Affiliations:** a Department of Cardiology, School of Medicine, University of Galway, Galway, Ireland; b CORRIB Research Center for Advanced Imaging and Core Laboratory, Galway, Ireland; c Centre for Cardiovascular Medicine and Devices, William Harvey Research Institute, Queen Mary University of London and Barts Heart Centre, London, UK; d Cleveland Clinic London, London, UK

**Keywords:** Effective orifice area, Mean pressure gradient, Myval, Sapien 3 Ultra Resilia, Transcatheter aortic valve implantation

## Abstract

• The Myval series are novel balloon-expandable transcatheter heart valves.
• The postprocedural effective orifice area and mean pressure gradient of the Myval series were compared with those of the Sapien 3 Ultra Resilia using published data.
• The Myval series seems to have effective orifice areas and mean pressure gradients comparable to those of Sapien 3 Ultra Resilia.

The Myval series are novel balloon-expandable transcatheter heart valves (THVs) that feature intermediate (21.5, 24.5, and 27.5 mm) and extra-large (30.5 and 32 mm) sizes in addition to the conventional sizes (20, 23, 26, and 29 mm). In the randomized LANDMARK trial, the Myval series (Myval and Myval Octacor) were compared with contemporary standard THV series (Sapien and Evolut) regarding the 30-day composite clinical outcome according to Valve Academic Research Consortium 3 and showed noninferiority.^1^^,^^2^ In the LANDMARK trial, the echocardiographic assessment by an independent echo core laboratory showed that Myval series had effective orifice areas (EOAs) equivalent to Sapien series (Sapien 3 [S3] and Sapien 3 Ultra [S3U]) in the 20 mm size but were significantly larger than Sapien series in the 23, 26, and 29 mm sizes.

Recently, the favorable hemodynamic outcomes of Sapien 3 Ultra Resilia (S3UR), the new iteration of Sapien series, have been published.^3^, ^4^, ^5^ The S3UR demonstrated significantly larger EOA than S3/S3U. The updated sewing method of the leaflets for the 20 and 23 mm sizes and anticalcification process applied to all sizes contribute presumably to the improved performance of S3UR. S3UR is available in the USA and Japan, and it recently received European Conformity mark. However, during the enrollment phase of the LANDMARK trial, it was not yet available in Europe. As a result, S3UR was not used in the LANDMARK trial.

To assess the hemodynamic performance of Myval series and S3UR, we compared postprocedural EOA between Myval series and S3UR using the published data: for Myval series, the data of the LANDMARK trial,^1^ and for S3UR, data of TVT^4^ and OCEAN-TAVI^5^ registries were used. In the LANDMARK trial, 30-day echocardiograms were analyzed by an independent core lab (CORRIB core lab, Ireland). In the TVT registry, site-reported echocardiograms at discharge, and in the OCEAN-TAVI registry, site-reported postprocedural echocardiograms were used.

The Figure shows the EOA and mean pressure gradient for each valve type and size. Myval seems to have EOAs and mean pressure gradients comparable to those of S3UR.

**Figure 1:**
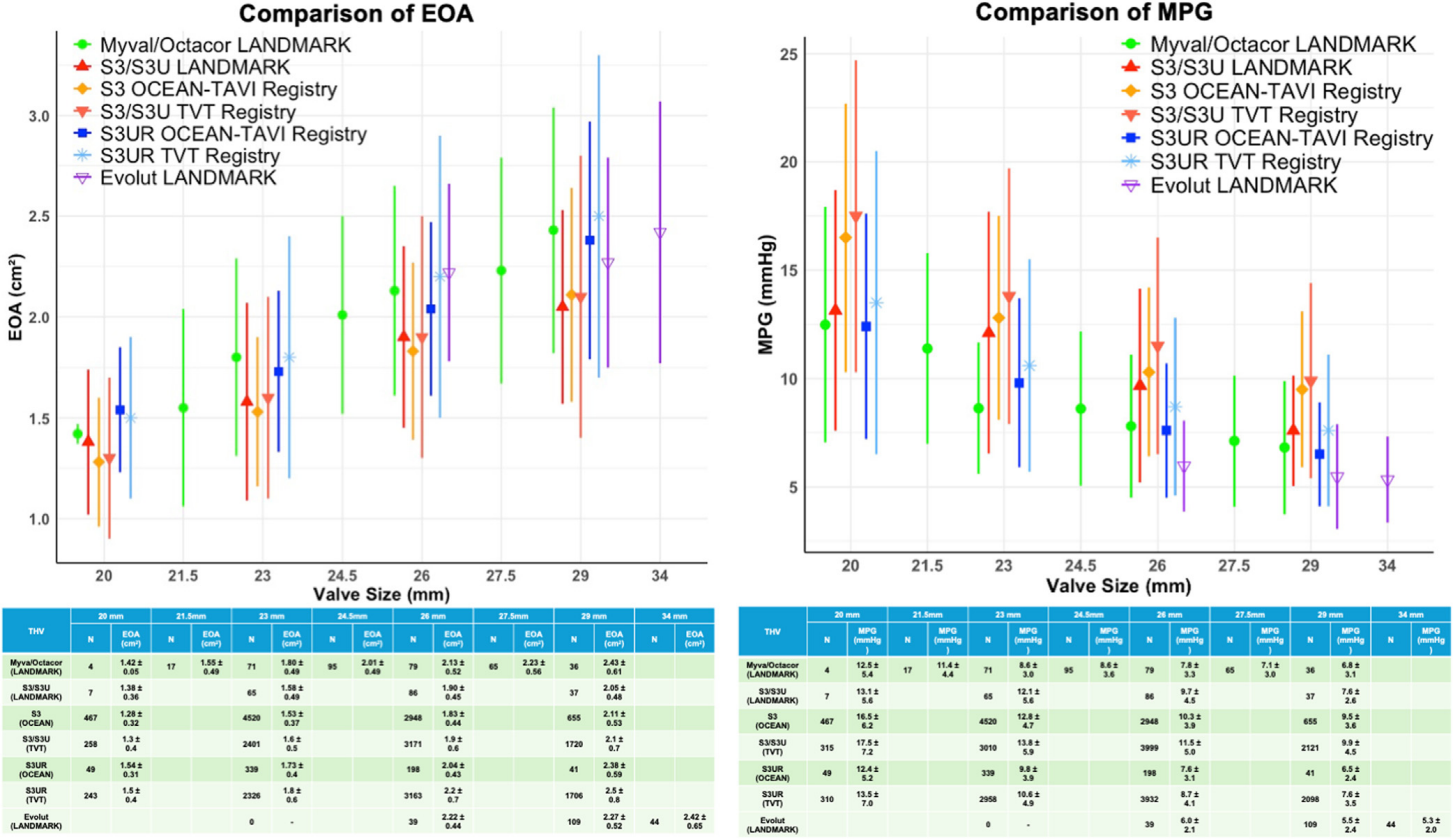
**Comparison of EOAs and MPGs.** The data for the Myval/Octacor, S3/S3U, and Evolut series from the LANDMARK trial, the S3 and S3UR from the OCEAN-TAVI registry, and the S3/S3U and S3UR from the TVT registry are presented. Evolut series in the LANDMARK trial includes Evolut R, Pro, Pro+, and FX. Abbreviations: EOA, effective orifice area; MPG, mean pressure gradient; S3, Sapien 3; S3U, Sapien 3 Ultra; S3UR, Sapien 3 Ultra Resilia.

We acknowledge that there are many limitations in this glimpse of hemodynamic comparisons, and it may be presumptuous to draw definitive conclusions. For example, overall, the small sample size of Myval series, particularly in 20 mm (n = 4), and the nonconsideration of other determinants of EOA, such as baseline anatomical characteristics, preclude statistical comparison at this stage. Furthermore, the manufacturer’s recommended aortic annulus areas for selecting THV size differ between Myval and Sapien series. Therefore, while the presented data provide an initial insight into hemodynamic performance of Myval series and S3UR, dedicated studies comparing the hemodynamics, as well as mid- and long-term clinical performance of these new technologies, are warranted.

## Ethics Statement

The LANDMARK trial was conducted in compliance with the International Council for Harmonisation of Technical Requirements for Pharmaceuticals for Human Use standards for clinical research. The data from TVT and OCEAN-TAVI registries were obtained from published sources.

## Funding

The LANDMARK trial is sponsored by Meril Life Sciences.

## Disclosure Statement

P.W. Serruys reports consulting fees from SMT, Novartis, Xeltis, Meril Life Sciences, and Philips. The other authors had no conflicts to declare.
